# From Spinal Central Pattern Generators to Cortical Network: Integrated BCI for Walking Rehabilitation

**DOI:** 10.1155/2012/375148

**Published:** 2012-01-04

**Authors:** G. Cheron, M. Duvinage, C. De Saedeleer, T. Castermans, A. Bengoetxea, M. Petieau, K. Seetharaman, T. Hoellinger, B. Dan, T. Dutoit, F. Sylos Labini, F. Lacquaniti, Y. Ivanenko

**Affiliations:** ^1^Laboratoire de Neurophysiologie et de Biomécanique du Mouvement, Université Libre de Bruxelles, CP 168, 50 Avenue F Roosevelt, 1050 Brussels, Belgium; ^2^Laboratory of Electrophysiology, University of Mons, 7000 Mons, Belgium; ^3^TCTS Lab, Faculty of Electrical Engineering, University of Mons, 7000 Mons, Belgium; ^4^Département de Neurologie, Hôpital des Enfants Reine Fabiola, Université libre de Bruxelles, 1050 Brussels, Belgium; ^5^Department of Neuromotor Physiology, Scientific Institute Foundation Santa Lucia 00179, Rome, Italy

## Abstract

Success in locomotor rehabilitation programs can be improved with the use of brain-computer interfaces (BCIs). Although a wealth of research has demonstrated that locomotion is largely controlled by spinal mechanisms, the brain is of utmost importance in monitoring locomotor patterns and therefore contains information regarding central pattern generation functioning. In addition, there is also a tight coordination between the upper and lower limbs, which can also be useful in controlling locomotion. The current paper critically investigates different approaches that are applicable to this field: the use of electroencephalogram (EEG), upper limb electromyogram (EMG), or a hybrid of the two neurophysiological signals to control assistive exoskeletons used in locomotion based on programmable central pattern generators (PCPGs) or dynamic recurrent neural networks (DRNNs). Plantar surface tactile stimulation devices combined with virtual reality may provide the sensation of walking while in a supine position for use of training brain signals generated during locomotion. These methods may exploit mechanisms of brain plasticity and assist in the neurorehabilitation of gait in a variety of clinical conditions, including stroke, spinal trauma, multiple sclerosis, and cerebral palsy.

## 1. Introduction

More than 10 million people in the world live with some form of handicap caused by a central nervous system (CNS) disorder. Although there has been a recent breakthrough obtained in one paraplegic patient by epidural electrical stimulation applied in the lumbosacral spinal cord [1], the generalization of this type of clinical rehabilitation is far from being applicable to all patients suffering from spinal cord injuries or other CNS movement disorders. Before the accident which resulted in injury at the C7/T1 level leading to complete paraplegia with some preservation of sensation, the subject reported in the Lancet was an athlete in extraordinary physical condition. Before epidural stimulation, this subject underwent numerous locomotor training sessions over a period of 26 months with no significant effect. It was only when a 16-electrode array was surgically placed on the dura (L1-S1 cord segments), allowing appropriate chronic electrical stimulation, that standing and stepping could be successfully induced. This latter performance is particularly relevant as since the very first step, human CNS must achieve a conservative (postural stability) and a destabilizing (dynamic control of the body and limbs for forward progression) function [2–5].

The fundamental background that has allowed the clinical success of Egderton's group is a good example of what is possible to accomplish when multidisciplinary basic and applied sciences work conjointly. Basic research was first carried out in animals in order to better understand the spinal cord networking. This permitted the discovery of a general structure called a central pattern generator (CPG) acting as a network of antagonist oscillators specifically dedicated to extensor or flexor muscles acting at the different joints [6, 7]. Although CPG exists in all invertebrate and vertebrate animals, it has not been studied much in mammals. The results obtained by Egderton's group [1] provide elegant evidence that the CPG exists in the human spinal cord. How the CPG integrates descending commands and sensory feedback is one of the main challenges in motor systems neuroscience [8–12]. In this context, experiments performed in animals [13] clearly indicate that combined approaches based on training technology including electrical, robotic, and pharmacological manipulations promote plasticity and functional recovery.

The present review addresses the perspectives offered by applying the concept of hybrid BCI recently developed by Pfurtscheller et al. [14] in rehabilitation of human walking. In this approach, at least two complementary BCI systems must work together in order to fulfill the following criteria: the signals must be directly related to brain activity, they must be treated in real time, and at least one type of brain signals must be intentionally modulated for a goal-directed behavior that includes feedback. We illustrate possible avenues for further developments with methodological and experimental examples from our present program initiated in 2010. In this development, we also propose parallel and/or convergent avenues for a hybrid BCI for walking rehabilitation. This is mainly motivated by the fact that it would not be currently realistic to expect a unique BCI procedure to reliably control such a complex behavior. The results reviewed here only represent first attempts which might pave the way for future developments.

## 2. What Are the Perspectives Offered by EEG for Walking Rehabilitation?

Over the last decade, EEG applied in the emerging field of neuronal oscillations [15] has provided new insights into the neurophysiological activity underlying sensorimotor rhythms. High-density EEG recording may overcome the limitations of the traditional brain imaging methods, such as confinement, immobility, and unnatural environment [16]. EEG is a unique noninvasive method allowing sufficient time resolution to record brain activity on the time scale of sensorimotor control exerted by the brain. To date, this modality has been the principal noninvasive source of physiological signals for BCI purposes. Surprisingly, the BCI control obtained with scalp-recorded EEG rhythms is situated in the same performance range in terms of speed and precision as the control obtained with intracortical ensemble of single neurons for nonlocomotor tasks [17].

## 3. EEG Recording during Locomotion: Difficulties and Expectations

Whatever the BCI detection system used, the search for a functional baseline remains a great challenge [18]. This necessarily implies the control of the resting or “default” condition of the awake brain. This default state cannot be regarded as “a simple resting state” but rather as a highly complex situation involving dynamic interplay between conscious and unconscious processes. Such a brain state can be considered as a transient equilibrium integrating many aspects of past experience for future prediction use [19]. This default mode of the brain is related to the concept of the “global work space” of consciousness [20] in which an “observing” or a “homunculus” function is exerted by the frontal pole of the brain on its own sensory influx [21]. In this framework, Malach's group has found with fMRI technique that a large part of the cortex consistently responded when subjects were exposed to an audiovisual movie providing a rich and multidimensional natural stimuli [22]. This “activated” pattern was at the same time accompanied by the presence of persistent “cortical islands” which failed to respond in a clear time-locked manner to the movie stimulation. These regions were not silent during movie watching, but they displayed a well-correlated spontaneous activity throughout the different “islands” forming a functional chain of an “intrinsic” system organized in complement to the “extrinsic” system that deals with processing of the sensory influx [23–25]. In order to overcome the default mode problem, vibrotactile and/or visual stimulation are used here in both training of the subject and during the actual operation of the BCI.

## 4. A Multiple Integrated Approach

**Figure 1:**
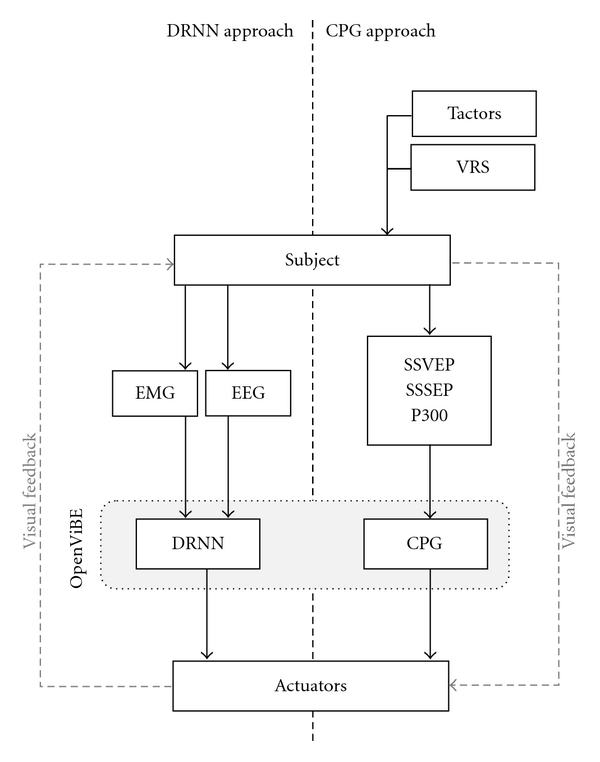
Research axes of MINDWALKER and BIOFACT projects. Parallel and convergent pathways using a dynamic recurrent neural network (DRNN, left part) and a central pattern generator (CPG, right part). The DRNN receives as input either the EMG signals from shoulder muscles mimicking the walking movement or the spontaneous EEG signals during walking. The CPG receives either steady-state somatosensory evoked potentials (SSSEPs), steady-state visual evoked potential (SSVEP), or classical P300 as starting signal. The SSSEPs are elicited by vibrotactile (tactors) stimulation on the foot sole mimicking walking patterns. A virtual reality stimulator (VRS) is used in order to generate an image of a walking mannequin to elicit SSVEP or to produce visual stimulation related to P300 speller.

Although locomotion is one of the most accessible rhythmical movements performed by humans, it is only recently that EEG studies related to locomotion were undertaken [26–28]. At the same time, we initiated the MINDWALKER and BIOFACT research projects in which we intend to develop an integrated BCI rehabilitation approach mainly based on EMG and EEG signals related to human locomotion. The following research axes (Figure 1) are developed in parallel: (1) identification and dynamic recurrent neural network (DRNN) recognition of the EMG patterns of the upper limb during treadmill walking, (2) identification and DRNN recognition of the EEG patterns during natural and treadmill walking, (3) development of an artificial CPG, (4) development of proprioceptive and visual stimulation for walking BCI, and (5) identification of walking imagination for BCI purpose. These five lines of research are progressively integrated into the OpenViBE environment [29] allowing additional virtual reality environment.

## 5. The DRNN Structure

The DRNN is a dynamic recurrent neural network which involves a looping mechanism (fully connected structure) which enables this network to learn and store information (memory). This equips the network with the ability to model complex situations with multiple influences. The DRNN is capable of modelling time varying input-outputs and has varying weights as well as varying time constants for the artificial neurons. The adaptive time constants make it dynamic. Additional information can be found in [30].

Our DRNN is governed by the following equation:

(1) $$T_{i}\frac{dy_{i}}{dt}=-y_{i}+F\left(x_{i}\right)+I_{i},$$

where *F*(*α*) is the squashing function *F*(*α*) = (1 + *e* ^−*α*^)^−1^, *y*_*i*_ is the state or activation level of unit *i*, *I*_*i*_ is an external input (or bias), and *x*_*i*_ is given by

(2) $$x_{i}=\underset{j}{\sum }w_{ij}y_{j},$$

which is the propagation equation of the network (*x*_*i*_ is called the total or effective input of the neuron, and *w*_*ij*_ is the synaptic weight between units *i* and *j*). The time constant *T*_*i*_ will act like a relaxation process. It allows a more complex dynamical behavior and improves the nonlinearity effect of the sigmoid function [31–33]. In order to make the temporal behavior of the network explicit, an error function is defined as

(3) $$E=\overset{t_{1}}{\underset{t_{0}}{\int }}q\left(y\left(t\right),t\right)dt,$$

where *t*_0_ and *t*_1_ give the time interval during which the correction process occurs. The function *q*(*y*(*t*), *t*) is the cost function at time *t* which depends on the vector of the neuron activations *y* and on time *t*. We then introduce new variables *p*_*i*_ (called adjoint variables) that will be determined by the following system of differential equations:

(4) $$\frac{dp_{i}}{dt}=\frac{1}{T_{i}}p_{i}-e_{i}-\underset{j}{\sum }\frac{1}{T_{i}}w_{ij}F^{\prime }\left(x_{j}\right)p_{j}$$

with boundary conditions *p*_*i*_(*t*_1_) = 0. After the introduction of these new variables, we can derive the learning equations:

(5) $$\begin{array}{c} \frac{\delta E}{\delta w_{ij}}=\frac{1}{T_{i}}\overset{t_{1}}{\underset{t_{0}}{\int }}y_{i}F^{\prime }\left(x_{j}\right)p_{j}dt,\\ \frac{\delta E}{\delta T_{i}}=\frac{1}{T_{i}}\overset{t_{1}}{\underset{t_{0}}{\int }}p_{i}\frac{dy_{i}}{dt}. \end{array}$$

The training is supervised, involving learning rule adaptations of synaptic weights and time constant of each unit (for more details, see [32]). This algorithm called “backpropagation through time” aims to minimize the error value defined as the differential area between the experimental and simulated output kinematics signals.

For certain noisy biological signals, such as the EEG, the results obtained using the procedure described above were not satisfactory. In some cases the convergence to a minimum error value was very slow or the learning process could lead to some bifurcations. In order to solve these problems, we brought two improvements to the DRNN training procedure: firstly, we introduced a convergence acceleration technique, and secondly we developed a technique to optimize the DRNN topology (i.e., the number of hidden neurons). The convergence acceleration technique we used is derived from Silva and Almeida [34]. In their method, they defined an adaptive learning rate *ε*_*ij*_ different for each interneuron connection, namely, for each synaptic weight and time constant. The acceleration is achieved by modifying the learning rate depending on the sign of the error function gradient. If the sign changes after iterating, it indicates that the learning went too far and that the learning step is too big. In this case, the learning rate is multiplied by a constant *d* (by default, *d* = 0.7). If there is no sign change after iterating, it means that the learning rate is too small. It is then multiplied by another constant *u* (by default, *u* = 1.3) in order to increase the step length and thus accelerate the search for the minimum error value. Formally, the algorithm is the following

- Small values are chosen for each *ε*_*ij*_.
- At the step *n*, the learning rate is defined as follows
- If
  (6) $$\frac{\partial E}{\partial w_{ij}}\left(n\right)\cdot \frac{\partial E}{\partial w_{ij}}\left(n-1\right)\geq 0.$$
  Then
  (7) $$\varepsilon_{ij}\left(n\right)=\varepsilon_{ij}\left(n-1\right)\cdot u.$$
  Else
  (8) $$\varepsilon_{ij}\left(n\right)=\varepsilon_{ij}\left(n-1\right)\cdot d.$$

(iii) The connections *w*_*ij*_ are incremented:
  (9) $$\Delta w_{ij}\left(n\right)={-\varepsilon }_{ij}\left(n\right)\cdot \frac{\partial E}{\partial w_{ij}}\left(n\right).$$

We observed that this method effectively accelerates the learning convergence, but it can also lead to an abnormal behavior, such as a monotonous increase of the error function along the number of learning steps. Therefore, a new procedure was developed checking at each learning step if the new learning rate *ε*_*ij*_ will not lead to an increase of the error function. In that case, learning rates are halved. For each step *n*, the mathematic expression is the following.

If

(10) E(n+1)>E(n).

Then

(11) $$\varepsilon_{ij}\left(n\right)=\frac{\varepsilon_{ij}\left(n\right)}{2}.$$

Thanks to this procedure, the error function now exhibits a much better behavior. Finally, in order to obtain the best possible results, the parameters that lead to the lowest error are stored along with the learning. Thanks to these enhancements, the DRNN has a higher probability of converging during the training phase.

Because the initial values of the parameters are random values, a global optimization of the topology is valuable. It consists in training a certain amount of different neural networks with a certain topology, and then, the same procedure has to be repeated with another DRNN topology.

## 6. Recognition of the Shoulder Muscles EMG Signals by a Dynamic Recurrent Neural Network Can Reproduce Lower Limb Locomotion in Human

**Figure 2:**
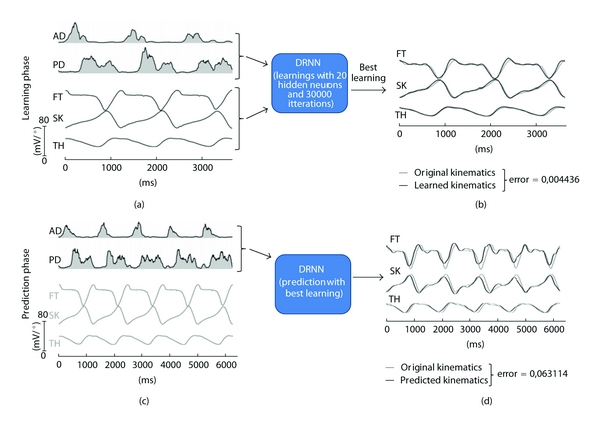
Kinematics of the lower limb predicted by a DRNN receiving shoulder muscles EMG signals. (a) During the learning phase, smoothed and rectified EMG signals of the anterior and posterior deltoid muscle (AD, PD) are used as input while the elevation angles of the feet (FT), the shank (SK), and the thigh (TH) are the desired outputs. (b) Superimposition of the real and simulated elevation angle curves. (c) During the prediction phase, unlearned EMG used as input to the DRNN. (d) Superimposition of the real and simulated elevation angle curves produced by unlearned EMG data.

We have previously demonstrated that a dynamic recurrent neural network (DRNN) is able to use multiple EMG bursts as inputs to reproduce lower limb movement in human locomotion [35]. In the present application we demonstrate that a DRNN is able to reproduce the elevation angles of the thigh, shank, and foot of human locomotion by means of only two EMG signals of the arm (rectified, filtered, and smoothed) recorded from the anterior and posterior deltoid muscles and used as DRNN input. For reaching this performance the DRNN comprises 20 fully connected hidden units. The training is supervised, involving the backpropagation through time algorithm. Subjects walked at different speeds (1.5, 3.0, 4.5, and 6.0 km/h) on a treadmill instrumented for contact force measurement. The elevation angles of the limb segments were computed using the positions of 23 passive markers disposed on the subject, determined thanks to six Infrared Bonita Vicon cameras. For each subject (*n* = 5), three continuous step cycles were used for the learning phase while five other unlearned step cycles were used for testing the DRNN's ability to reproduce the kinematics of lower limb locomotion.  Figure 2 shows that this approach is successful for producing lower limb kinematics by using only two EMG signals recorded at the anterior and posterior deltoid muscles. After the training (Figure 2(b)), the DRNN output signals almost perfectly fitted the real kinematics (error ~0.004). Moreover, this learned DRNN was able to produce very similar kinematics patterns (error ~0.06) by means of unlearned EMG inputs during the prediction phase (Figures 2(c) and 2(d)). 

The rhythmic bursting activity of the upper and lower limb muscles associated with human walking is, in some instances, the peripheral reflection of the temporal organization of muscle activation generated by the central neural structures. Indeed, the agonist-antagonist organization of the muscles and the reciprocal mode of command are represented by the reciprocal activity of a subset of cortical neurons correlated inside of cortical maps [36–38]. Of relatively smaller amplitude, the EMG burst of the shoulder muscles is in perfect reciprocal activation and in synchrony with the lower limb muscles bursting pattern in human walking [39]. Namely, the posterior deltoid muscle produces discharges in synchrony with the soleus muscle of the ipsilateral limb, facilitating the forward propulsion of the body after the push-off. In a reciprocal way, the anterior deltoid muscle produces discharges in phase with the tibialis anterior muscle, controlling the reaching phase of weight bearing. At present we are able to use these EMG signals in real time for producing the 3 elevation angles of the lower limb segments. This can be accomplished when the subjects are in seated position. Combined with other BCI procedures, this new task-dynamics recognition of the DRNN has implications for the development of diagnostic tools and prosthetic controllers.

## 7. A CPG Model between the EEG Signals and Mechanical Actuators

It is established that locomotion is governed by a hierarchical system [6, 7]. At the lowest level of this system is the CPG in the spinal cord. Inside the CPG, the motoneurons, which are the sole output unit of the spine, are coordinated by reciprocal inhibitory connections that can generate periodic patterns whose frequencies are controlled by the brain. The CPG mechanism has inspired the field of robotics, particularly in the development of small autonomous walking robots, from multilegged insect-like robots to humanoids [40] and active prostheses based on motion detection [41].

In this part inspired from [42], we review the development of a programmable CPG (PCPG) [43] capable of being easily driven by current BCI technology [44, 45]. A PCPG algorithm is able to generate any periodic pattern after a learning step. The interests of such a system lie in the simple parameterization learning and in the controllable aspect of the learned parameters, namely, the pattern magnitude and frequency. A modification of one of these parameters will lead to a smooth transition of the PCPG output. This is a particularly interesting feature, which is especially important for prosthesis applications and their actuators. Based on a PCPG learned at a medium speed, that is, 3 km/h, by tuning frequency and magnitude, we were able to adapt speed continuously according to the user's intent [46]. This means that, given a high-level command such as a P300-related order, the artificial actuator will execute all the low-level commands, for example, generate kinematics thanks to the PCPG and manage feedback control. Although special focus is on the coupling between several PCPGs, for example, between foot, shank, and thigh angles of elevation, particular attention was given on the control of the foot elevation angle which is known to be the most complex signal. A PCPG is a kind of Fourier series decomposition and is composed of several adaptive oscillators. As defined in Righetti et al. [43], this algorithm is governed by the following equation system:

(12) $$\begin{array}{cc} & {\dot{x}}_{i}=\gamma \left(\mu -r_{i}^{2}\right)x_{i}-\omega_{i}y_{i}+\epsilon F\left(t\right)+\tau \sin\left(R_{i}-\phi_{i}\right),\\ & {\dot{y}}_{i}=\gamma \left(\mu -r_{i}^{2}\right)y_{i}+\omega_{i}x_{i},\;\\ & {\dot{\omega }}_{i}=-\epsilon F\left(t\right)\frac{y_{i}}{r_{i}},\\ & {\dot{\alpha }}_{i}=\eta x_{i}F\left(t\right),\\ & {\dot{\phi }}_{0}=0,\\ & {\dot{\phi }}_{i}=\sin\left(R_{i}-\mathrm{sgn}\left(x_{i}\right)\cos^{-1}\left(-\frac{y_{i}}{r_{i}}\right)-\phi_{i}\right),\;\forall i\neq 0.\\ & \text{With}\\ & R_{i}=\frac{\omega_{i}}{\omega_{0}}\mathrm{sgn}\left(x_{0}\right)\cos^{-1}\left(-\frac{y_{0}}{r_{0}}\right),\\ & F\left(t\right)=P_{\text{teach}}\left(t\right)-\overset{N}{\underset{i=0}{\sum }}\alpha_{i}x_{i}. \end{array}$$

**Figure 3:**
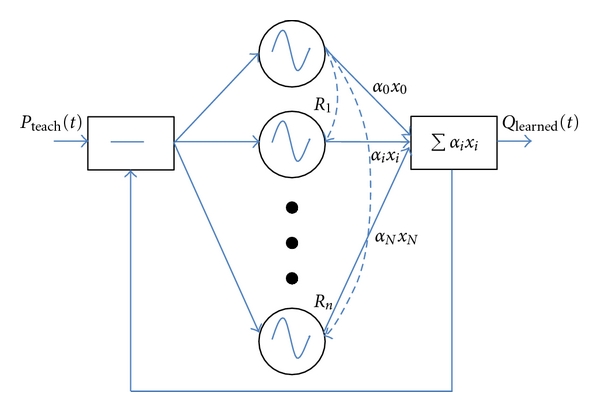
The PCPG is able to learn the frequency components of a periodic signal as well as the various phases and magnitudes. One major interest of PCPGs is the possibility to modify a learned pattern in amplitude or frequency in a smooth way. This figure is adapted from Righetti et al. [43].

As depicted in Figure 3, oscillators are coupled. The instantaneous phase of the fundamental oscillator *R*_0_ is scaled at *ω*_*i*_ through *R*_*i*_ and the phase difference with the fundamental oscillator is given by *ϕ*_*i*_. They are composed of adaptive magnitude coefficients *α*_*i*_ and frequency parameters *ω*_*i*_  (*r*_*i*_ = (*x*_*i*_^2^+  *y*_*i*_^2^)^1/2^ · *μ*) has a role of normalization of the learned pattern. The other parameters *γ*, *ϵ*, and *τ* aim at accelerating the convergence while limiting stability problems [43]. The *Q*_learned_(*t*) signal resulting from the sum of oscillator outputs is compared to the *P*_learned_(*t*) walking pattern target and the error value *F*(*t*) is computed. Throughout the learning step consisting of integrating the differential equations by a 4th-order Runge-Kutta method with a fixed step size, all the parameters of the PCPG are modified in order to minimize  *F*(*t*). When this learning step is finished, *F*(*t*) is close to zero and the system is generating the right pattern at the *Q*_learned_(*t*) output.

The PCPGs' properties make them suitable for trajectory generation in robotics and also for prosthesis applications. In fact, the pattern learned by a PCPG can be easily controlled in magnitude and in frequency thanks to a simple linear change of the *ω* and *α* vectors representing the *ℜ* ^*N*^ PCPG states (*N* is the number of oscillators). This linearity leads to a smooth change of the global system behavior. For instance, if the *ω* vector is divided by two, the underlying frequency of the standard temporal pattern is divided by two. The same effect occurs for the *α* vector. Finally, as proposed in Righetti et al. [43], it is possible to couple several PCPGs thanks to equations of coupling between the fundamental oscillators of each PCPG and the learning rules for the phase difference are defined as

(13) $$\begin{array}{c} \;{\dot{x}}_{0,k}=\left(\mu -r^{2}\right)x_{0,k}-\omega_{0,k}y_{0,k}+\tau \sin\left(R_{0,k}-\phi_{0,k}\right),\\ {\dot{\phi }}_{0,k}=\sin\left(R_{0,k-1}-R_{0,k}-\phi_{0,k}\right), \end{array}$$

where (0, *k*) denotes the first oscillator of the *k*th PCPG.

The originality of this PCPG is to generate walking patterns in a way differing from the bipedal robots described in the literature, which consists of walking as far as possible without taking into account the potential patient. Indeed, one of the main goals in prosthetics is to provide the user with the most comfortable walk possible. Therefore, at each step, the studied pattern should be adapted in terms of frequency and magnitude, that is, the stepping frequency and stride-related length between two heel strikes, respectively, whatever is the walking speed.

**Figure 4:**
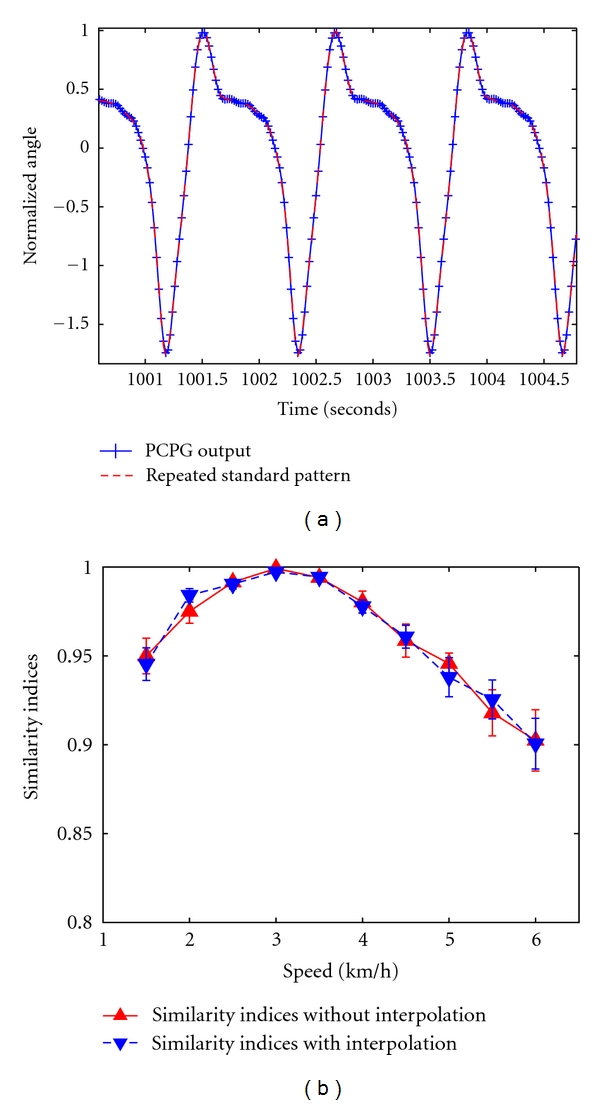
PCPG performance as a function of the walking speed. (a) Superimposition of the real foot elevation angle (red) and the PCPG output (blue) by means of 7 oscillators. (b) The difference between SI values obtained with and without the interpolation is not significant. In this case, error bars are standard errors (modified from Duvinage et al. [46]).

In order to train the PCPG for each subject, three standard walking patterns were needed. These temporal patterns consist of the angle of elevation of the foot, the shank, and the thigh of a healthy subject walking on a treadmill at 3 km/h, a typically medium speed for humans. Each standard pattern is a kind of an average pattern along the 60-second recordings. After determining and normalizing these standard patterns, the PCPG was trained using the procedure previously described.  Figure 4(a) shows the PCPG's ability to reproduce the foot elevation angle using 7 oscillators. Kinematics data were recorded in different subjects (*n* = 7) at 10 different speeds, from 1.5 to 6 km/h, at steps of 0.5 km/h. For a specific type of angle of elevation, the normalized and centered pattern learned by the PCPG for the 3 km/h speed was generated for all the other speeds.

To prove the relevance of this approach, a Similarity Index (SI) [48] was assessed between the PCPG output *f*_1_(*t*) and the standard walking pattern *f*_2_(*t*) at each speed to show the true potential of this method and the result of the mathematical link between PCPG parameters and speed.

This index is defined as

(14) $$\text{ SI}=\frac{\int_{-T/2}^{T/2}f_{1}f_{2}{\left(t\right)}^{2}dt}{{\left[\int_{-T/2}^{T/2}f_{1}{\left(t\right)}^{2}dt\int_{-T/2}^{T/2}f_{2}{\left(t\right)}^{2}dt\right]}^{1/2}},$$

where *T* is the period of the limit cycle, *f*_1_(*t*) and *f*_2_(*t*) being synchronized; that is, the matching between both patterns is based on the maximum of each pattern. Note that if both functions are identical, SI = 1. 

Figure 4(b) shows that SI values without interpolation are very good but show a logical degradation for speeds situated at both sides from the PCPG-learned speed. Regarding the interpolation, the impact of the dissimilarity increase is clearly negligible. An alternative to improve this procedure which relies on a single PCPG could be to manage a multi-PCPG system at a multi-interpolation level; each PCPG will model a typical range of speeds with its own interpolation, for example, 0.5–2 km/h where SI are sufficiently high compared to the level of requirements. The merging of those PCPGs would be used to model as perfectly as possible real walk while making the change of PCPG as smooth as possible. Although results are presented for the foot elevation, similar results were obtained for the other elevation angles. This approach can be easily extended to a full lower limb prosthesis in its principles [46]. Finally we recently demonstrated that a mathematical link based on polynomial interpolation is sufficient to control the PCPG parameters along the speed and that human walk can be learned by a PCPG controlling different walking speeds. Obviously, at constant speed, gait cycles are not perfectly identical [49]. This fact and numerous perturbations can provoke phase mismatch between the perfectly periodic PCPG output and the real gait pattern in addition to change in frequency. If this mismatch is too important, the subject has to compensate for it leading to a nonnatural gait.

The aim of the phase-resetting is to pave the way to allow the orthosis to adapt to the patient as quickly and smoothly as possible aiming at increasing the subject comfort. The phase-resetting consists in resynchronizing the PCPG state according to special events. Therefore, the PCPG could be phase reset on the heel strike to allow the system to recover the correct phase in a smooth way at the time of the toe off. Up to now, two approaches are available: a hard phase resetting, which allows to instantaneously recover the actual gait phase at the price of uncomfortable kinematics discontinuities, and a soft phase-resetting, which allows to recover the phase in a smooth but slow way. Further details are available in [46, 51].

## 8. EEG and DRNN during Walking

**Figure 5:**
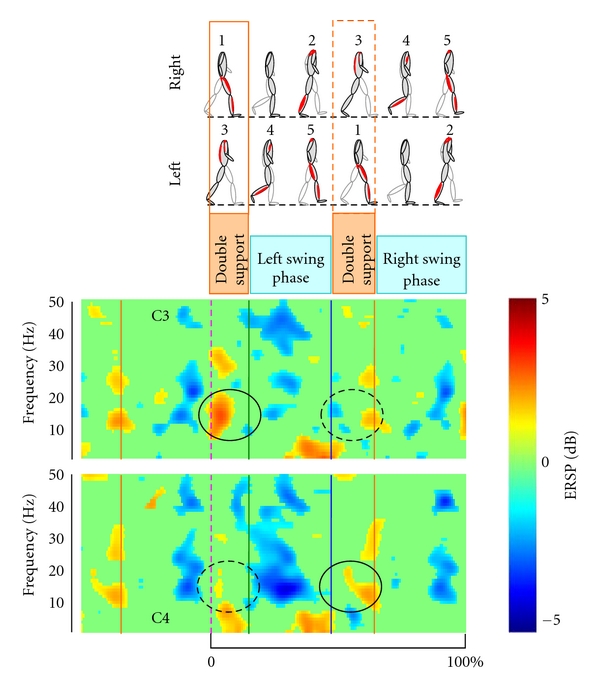
Event-related spectral perturbation (ERSP) during walking cycle recorded in C3 and C4 electrodes for one subject. The stripped lines indicate the right heel strike event upon which the averaging was triggered. The power increase is represented in red color and the power decrease in blue color.

EEG was recorded using a 128-electrode cap connected to the ANT acquisition system (Advanced Neuro Technology, ANT, Enschede, The Netherlands) digitizing the signals at 2048 Hz. Left ear was chosen as reference. In parallel, the kinematics of the lower limb movements was recorded using a system of six infrared cameras (Bonita, Vicon, Los Angeles, CA, USA) determining at a frequency of 100 Hz, the *x*, *y*, and *z* coordinates of 23 passive markers placed on the subjects. In the first step, kinematics data and EEG signals were synchronized. The elevation angles of both feet were then computed as a function of time. A peak-detection algorithm was used to localize precisely the successive heel strike and push off events. EEG signals were processed using the EEGLAB toolbox [52]. Prior to performing independent component analysis (ICA), we high-pass filtered the EEG signals above 1 Hz and the time periods of EEG with artifact were rejected on amplitude criteria. Then we have performed ICA method for extracting the component related to eye movement which is mainly expressed in frontal area.  Figure 5 illustrates the ERSP analysis of the EEG recorded in C3 and C4 channels during 50 walking cycles. For this subject, the upper alpha and beta bands presented a significant power increase during the stance period. This was clearly showed when the averaging was triggered when the heel of the leading foot was contacting the ground (0%, dotted line) and the trailing foot was pushing off (green line). This power increase was more important at the C3 electrode for the right heel strike event than for the left heel strike. This situation was reversed at the C4 electrode. Interestingly, the left swing phase was accompanied by a significant alpha-beta power decrease in C4 and a gamma power decrease in C3. These ERSP rhythmic modulations highlight the EEG involvement in human walk. These results seem coherent with other publications [11, 27]. The present EEG results during treadmill locomotion show that different rhythms (alpha-beta-gamma) are specifically involved in the control of the walking pattern and that it is possible to extract EEG signals from the sensorimotor cortex controlling the contralateral foot placement. This confirms the recent studies of Gwin et al. [27] and motivated our actual and future work in the search of EEG signals related to walking imagery. 

**Figure 6:**
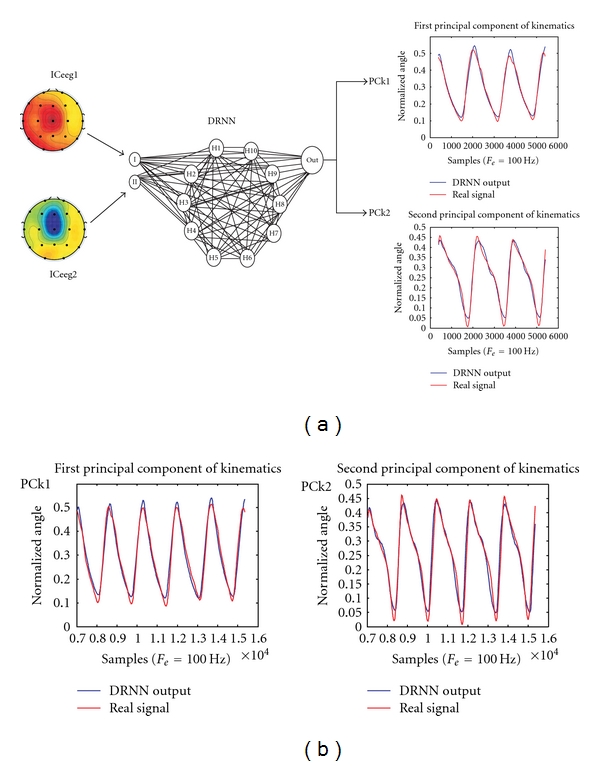
DRNN learning (a) and simulation (b) phases where two independent components (ICeeg_1-2_) were used as input to the DRNN while the two principal components (PCk_1-2_) of the 3 elevation angles foot, shank, and thigh of one lower limb kinematics were used as desired output (modified from [47]).

Based on this EEG-related walking modulation, different attempts were done in order to introduce the EEG signals as input of the DRNN [47]. For the best subject we have, we describe here the approach using the two most representative independent components (ICeeg_1-2_) of the sensorimotor cortex as input for a DRNN learning identification toward the two principal components (PCk_1-2_) of the 3 elevation angles foot, shank, and thigh of one lower limb kinematics. This was motivated by the recent fMRI data indicating a cortical generator around Cz (bilateral central region Ba4 and Ba3) [53].  Figure 6(a) illustrates the success of the learning phase of the DRNN which produced a very good reproducibility of the 2 kinematics PC (Figure 6(a), see the superimposition of the real PC in red and the DRNN output in blue). After this learning (Figure 6(b)), the sending of new (unlearned data) ICA-related EEG signals corresponding to walking pattern in the learned DRNN produces reasonable signal output (blue line) close to the 2 PC of the real kinematics (red line). Although the performance was less strong for other subjects, the gait rhythm was correctly extracted [47].

**Figure 7:**
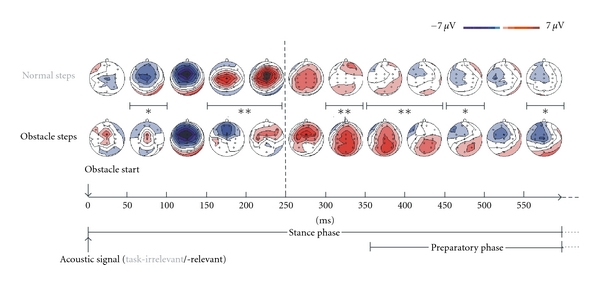
EEG activity during normal and obstacle steps performed on a treadmill. Grand averages of the initial EEG activity and topography (32 EEG electrodes placed on the scalp) from 12 subjects. The stance and swing phases were determined by the time period where all 12 subjects were on stance and swing (i.e., the individually shortest stance and swing phases, resp.). In normal steps a task-irrelevant and in obstacle steps a task-relevant acoustic signal was delivered at the onset of the right stance phase. Significant differences are indicated by asterisks (*P < 0.05, **P < 0.01) (modified with permission of Figure 3 from Haefeli et al. [11]).

 EEG activity can also be used as a signal reflecting interaction between brain and spinal neuronal activity during complex locomotor tasks. For instance, Haefeli et al. [11] recently confirmed our results on the localization of the EEG activity during normal walking (Figure 7) and showed that stepping over an obstacle is associated with enhanced EEG signal in the prefrontal cortex (Figure 7). Furthermore, superposition of an invariant locomotion timing pattern with voluntary activation timing is consistent with the proposal suggesting that compound movements are produced through a superposition of motor programs [54]. Thus, the EEG can be potentially used to feed DRNN for characterizing the interaction of locomotion with voluntary movements.

## 9. Vibrotactile-Evoked Potentials

**Figure 8:**
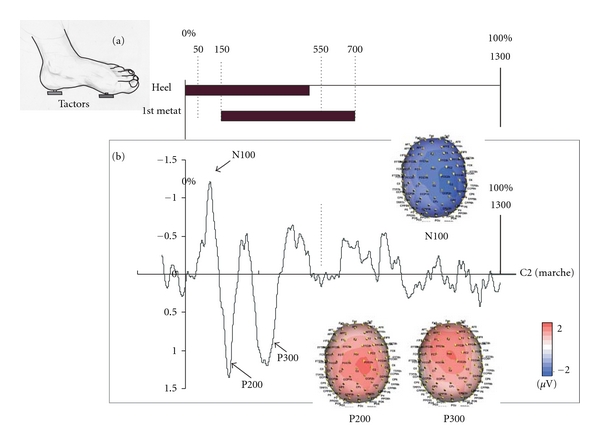
Vibrotactile stimulation. (a) The time profile of the vibrotactile stimulation provided by heel and first metatarsal tactors is shown. The timing and the duration of vibration correspond to a gait cycle at 3 km/h speed [50]. (b) The evoked potential showing 3 main components (N100, P200, and P300) recorded at C2 electrode. The color maps show the distribution of these 3 evoked components. The C-2 Tactor is a linear actuator that has been optimized for use against the skin. The C-2 Tactor incorporates a moving “contactor” that is lightly preloaded against the skin. When an electrical signal is applied, the “contactor” oscillates perpendicular to the skin, while the surrounding skin area is “shielded” with a passive housing. Thus, unlike most vibrational transducers (such as common eccentric mass motors that simply shake the entire device), the C-2 provides a strong, point-like sensation that is easily felt and localized. For optimum vibrotactile efficiency, the C-2 is designed with a primary resonance in the 200–300 Hz range that coincides with peak sensitivity of the Pacinian corpuscle, the skin's mechanoreceptors that sense vibration. The subjects are seated with both feet on the floor, wearing a sandal on the left foot with a first tactor at the level of the heel and a second tactor at the level of the head of the first metatarsal. The stimulation of each tactor is made at a frequency of 300 Hz.

In order to increase the understanding of EEG signals related to locomotion and the influence of afferent input into the cortical activities, we first studied the evoked potential induced by the vibrotactile stimulation of the foot sole. The purpose is to simulate the real mechanical stimulation exerted by the ground on the plantar foot corresponding to gait cycles. It is known that sustained attention to a body location results in enhanced processing of tactile stimuli presented at that location compared to another unattended location [55]. In our protocol (Figure 8(a)), the attention is directed to the plantar sole stimulation given by the two tactors mimicking the gait pace. By this procedure we expect to characterize an evoked potential related not only to the peripheral vibration by itself but also to the recognition of the gait sensation. Figure 8(b) illustrates in one representative subject the evoked potential corresponding to one gait cycle. Three main components are evoked, the N100, P200, and P300. The first two components were localized in the contralateral sensorimotor cortex while the P300 was recorded more on the midline of the frontal cortex.

While standard evoked potentials (SEPs or VEPs) may be used for detecting isolated components as the P300 and the relative gating of some peculiar components [56], the steady-state evoked potential (SSVEP or SSSEP) may offer an interesting alternative BCI in subjects who have trouble producing the EEG activity necessary to use an ERD BCI [57, 58]. The present SSSEP produced by the foot tactors may offer a controlled “extrinsic” system upon which high-level BCI command could be organized as recently demonstrated for the SSVEP, allowing a very high information transfer rate [59]. The “intrinsic” attractor state can also modify the brain's ability to analyze environmental information and to organize final action. Interestingly, the prefrontal cortex engaged in self-related introspective processes is inhibited during sensorimotor processing [60]. This antagonistic and patchwork-like organization largely complicated the definition of a functional baseline because it necessitates a previous knowledge of the “extrinsic” and “intrinsic” networks' localization during a particular task. As volition is central for the initiation of a BCI process, a better understanding of the “intrinsic” system behavior is urgently required. However, the tendency of different human individuals to present similar patterns of brain activation when they are confronted to natural visual scenes [61] encourages the utilization of ecological virtual reality stimulator for deciphering the dynamical dialogue between intrinsic and extrinsic neural network in real space work. Ideally, the baseline should be better defined as a physiological state, rather than just the arbitrary period preceding the task [18, 19].

## 10. Practical and Future Aspects of the Integrated Approach

The present integrated approach allows us to propose different practical strategies for rehabilitation of the subject depending on the specificities/clinical requirements of each individual subject. The first possible clinical application is the task-dynamics recognition of the EMG-DRNN which has implications for the development of diagnostic tools (comparative analysis between DRNN identification of normal and pathological motor strategies [5]) and prosthetic controllers. In patients with paraplegia, for example, with only the recording of the shoulder muscles EMG, the DRNN could be able to produce 3 kinematics signals of the lower limb allowing exoskeleton activation for locomotion.

As the EEG-DRNN seems to be able to produce walking kinematics during actual locomotion, the next step is to prove that this can be done with EEG signals during imagination of walking. In this context, the vibrotactile and the visual stimuli could reinforce the sensation of walking and facilitate the identification of pertinent EEG components. Another possible strategy involves combining the EMG-DRNN and the EEG-DRNN, working in a parallel or a hybrid manner. 

The merging of multiple PCPGs would be used to model, as perfectly as possible, real walk while making the change of PCPG as smooth as possible. Moreover, as BCI for walking rehabilitation is far from working perfectly, a confidence level of the command could be derived and integrated in the speed parameter change. Considering that an *accelerate* command increases the actual speed of 0.5 km/h by default, if the decision is uncertain, for example, reliable at 75%, 75% of the speed increase can be actually performed thanks to the parameter interpolation. With this approach a classical BCI paradigm (i.e., P300 speller detection) can be used to provide a high-level command for walking [62, 63].

Finally, the DRNN output can be used as a trigger or control signal for the PCPG mimicking the biological control pathways exerted by the cortex on the spinal CPGs. Indeed, the DRNN could convert the EEG signal into a gait phase, that is, where the patient is in the gait cycle. The gait phase could control the PCPG in frequency by computing the derivative of the phase. Moreover, the PCPG could be reset by this phase information.

## 11. Conclusion

The present proposal for hybrid BCI system including walking imagery inducing artificial movement of the actuators supporting human body must also take into account the emerging pattern of collective network. Indeed, brain oscillations are inherently linked to the neuronal behavior that gave rise to it and, in turn, definitely influence the global behavior of the system. This also implies a high level of control of “state transitions” in EEG activity which could be facilitated by using the modulation of the steady-state evoked response. It was demonstrated that each transition began with an abrupt phase resetting followed by resynchronization, spatial pattern stabilization, and increase in global pattern amplitude [64]. A hybrid BCI system including the different research axes presented here in their preliminary states must be able in the close future to help with walking rehabilitation.
